# Lower T Regulatory and Th17 Cell Populations Predicted by RT-PCR-Amplified *FOXP3* and *ROR*γ*t* Genes Are Not Rare in Patients With Primary Immunodeficiency Diseases

**DOI:** 10.3389/fimmu.2020.01111

**Published:** 2020-06-25

**Authors:** Wen-I Lee, Jing-Long Huang, Syh-Jae Lin, Kuo-Wei Yeh, Li-Chen Chen, Liang-Shiou Ou, Tsung-Chieh Yao, Tang-Her Jaing, Ying-Fan Shih, Chao-Yi Wu

**Affiliations:** ^1^Primary Immunodeficiency Care and Research (PICAR) Institute, Taoyuan, Taiwan; ^2^Division of Allergy, Asthma, and Rheumatology, Department of Pediatrics, Chang Gung Memorial Hospital, Chang Gung University College of Medicine, Taoyuan, Taiwan; ^3^Department of Pediatrics, New Taipei Municipal TuChen Hospital, Taoyuan, Taiwan; ^4^Division of Hematology/Oncology, Department of Pediatrics, Chang Gung Memorial Hospital, Chang Gung University College of Medicine, Taoyuan, Taiwan

**Keywords:** primary immunodeficiency, T regulatory cell, Th17 cell, autoimmune disorder, recurrent infection, Foxp3, RORγt

## Abstract

Deficiencies in T regulatory (Treg) and Th17 cells attenuate peripheral tolerance and the IL-17 family of cytokines, contributing to autoimmune disorders and opportunistic (fungal) infections, respectively. Because of limited blood samples from patients with primary immunodeficiency diseases (PIDs), a positive correlation/linear relationship between Treg and Th17 cells and their respective expressions of transcription factors forkhead box P3 (FOXP3) and retinoic acid-related orphan receptor γ (RORγt) by real-time PCR (RT-PCR) amplification, was used to predict the percentages of Treg and Th17 cells in peripheral blood. Compared to glyceraldehyde 3-phosphate dehydrogenase (GAPDH) expression, the percentages of Treg and Th17 cells were calculated as the linear relationship to the 2^−ΔCT^ value (cycle threshold). Among 91 PIDs patients, 68 and 78 had predicted Treg and Th17 percentages below 5% of the normal ranges (0.859 and 0.734%, respectively), which expanded different categories beyond obvious T cell deficiency. Notably, FOXP3 was undetectable in one patient (CVID), RORγt was undetectable in six patients (one CVID, one CID, two neutropenia, one WAS, and one CMC), and both were undetectable in four patients (two SCID, one STAT1, and one periodic fever). In contrast, two patients with auto-IFNγ antibodies had increased susceptibility to intracellular mycobacterial infections, interrupted Th1 development and subsequent elevation in the Th17 cells. Both predicted Treg and Th17 percentages in the PIDs patients were more independent of age (months) than in the controls. The predicted Th17/Treg ratio in the PIDs patients, overall, was lower than that in the healthy controls (0.79 ± 0.075 vs. 1.16 ± 0.208; *p* = 0.038). In conclusion, lower predicted Treg and Th17 cell populations calculated by RT-PCR-amplified FOXP3 and RORγt in PIDs patients at diagnosis can explain the higher potential phenotypes of autoimmune disorders and opportunistic infections, although effective interventions in the early stage might have prevented such phenotypic development and caused a statistical bias in the comparisons.

## Introduction

Patients with primary immunodeficiency diseases (PIDs) have genetic defects or etiologies of immune dysfunction and recurrent (opportunistic) infections, and advanced medical interventions can improve their survival (1–3). However, due to the longer life expectancy, these patients have a greater possibility of developing autoimmune disorders (4, 5). Insufficient T regulatory (Treg) cells attenuate tolerance and lead to the release of autoantibodies, thereby causing autoimmune disorders (6, 7). Th17 cells produce the IL-17 family of cytokines on epithelium, which resist dermal opportunistic infections and construct intact intestinal barriers. Th17 cell dysfunction is associated with chronic muco-cutaneous candidiasis (CMC) (8) and celiac-like chronic diarrhea (9).

Reacting with the antigen/major histocompatibility complex (MHC) and CD3/CD28/co-stimulatory ligands (B7-1/B7-2) (10), naive T cells can proliferate and differentiate into Treg and Th17 cells through the transcription factor's forkhead box P3 (FOXP3) and retinoic acid-related orphan receptor γ (RORγt), respectively. Mutations of *FOXP3* have been shown to cause the Scurfy phenotype of IPEX syndrome (immunodeficiency, poly-endocrinopathy, enteropathy, and X-linked) in mice and humans (11, 12). IPEX-like syndrome can occur in patients with defective IL-2 signaling [due to mutations of the *CD25* (IL2Rα) and *STAT5b* gene] (13, 14), insufficient IL-10 signaling [due to mutations of the *IL10* and *IL10R* genes] (15–17), and CTLA4 inhibition (due to mutations of the *CTLA4* (18–20) and *LRBA* genes) (21, 22) all of which attenuate Treg suppression. A reduced RORγt expression and the consequent impairment of Th17 cell generation (23) have been described in patients with APECED (Autoimmune PolyEndocrinopathy with Candidiasis and Ectodermal Dystrophy) due to mutations of the *AIRE* (AutoImmune REgulator) gene (24) and hyper-IgE recurrent infection syndromes (HIES) due to mutations of the signal transducer and activator of transcription 3 (*STAT3*) (25–28), tyrosine kinase 2 (*TYK2*) (29, 30), dedicator of cytokinesis 8 (*DOCK8*) (31, 32), and *RORC* genes (33). Consequently, these T cell immunodeficiencies can prevent the development of Treg and Th17 cells and contribute to autoimmune disorders and opportunistic infections.

The aim of this study was to investigate the use of a linear relationship to estimate Treg and Th17 cell proportions by quantitative real-time PCR (RT-PCR) amplification of FOXP3 and RORγt from residual complement DNA after genetic analysis. We speculated whether the estimated percentages of Treg and Th17 cells could reflect immune status at diagnosis and correlate to the likelihood of developing autoimmune disorders and acquiring opportunistic infections.

## Methods

### Patients

Since 2003, 727 individuals (573 suspected cases and 154 related persons) have been referred to the Primary Immunodeficiency Care and Research (PICAR) Institute for molecular/genetic diagnosis of PIDs. Two hundred and fifty patients were identified as having PIDs (Supplemental Table 1). According to the 2019 updated PIDs categories (34), “combined immunodeficiencies with associated or syndromic features” (previously termed “well-defined syndromes with immunodeficiency”) (*n* = 89) were the most common PIDs in Taiwan, followed by “predominantly antibody deficiencies” (*n* = 57), “combined T- and B-cell immunodeficiencies” (*n* = 40), congenital defects of phagocytes (*n* = 35), complement deficiencies (*n* = 15), diseases of immune dysregulation (*n* = 6), defects in innate immunity (*n* = 2), auto-inflammatory disorders (*n* = 2), and copies of PIDs (*n* = 4). Bone marrow failure was first added as a category in the 2019 update, however no patient in this category is enrolled at present.

The Chang Gung Human Investigation Committee approved this study. The patients' parents or guardians provided written and verbal informed consent. Basic immunologic functions (3, 35) and candidate genes were sequenced from complementary DNA synthesized from RNA isolation and confirmed again by genomic DNA as previously described (36).

### Evaluation of FOXP3 and RORγt Expressions by Real-Time PCR

Total RNA was isolated from PBMCs with TRIzol (Invitrogen, Carlsbad, CA) (36). The amounts of FOXP3 and RORγt were simultaneously detected using an RT-PCR assay on a 7500 Fast Real-Time PCR system (Applied Biosystems). Briefly, 20 μl cDNA was synthesized from 2 μg RNA. Then, 3 μl cDNA was mixed with 10 μl ABI Master Mix, and 1 μl Primer-Probe was run in a final volume of 20 μl (ABI Prism 7900, Applied Biosystems). The following primer and probe sets were purchased from Applied Biosystems: FOXP3 (Hs01085834_m1) and RORγt (Hs01076112_m1), with GAPDH (Hs99999905_m1) as the internal control. The program for the three genes was set-up with the same constant protocol: a first step at 50°C for 2 min, initial heating at 95°C for 10 min, 50 cycles of denaturation at 95°C for 15 s, annealing at 60°C for 1 min, and elongation also at 60°C for 1 min.

Samples were assayed in duplicate. ΔCT-FOXP3 and ΔCT- RORγt (CT: cycle threshold; ΔCT = CT-FOXP3 or CT- RORγt minus CT-GAPDH) were calculated for each genetic expression, while *GAPDH* was used as the internal control housekeeping gene. Relative fold expression changes of the *FOXP3* and *RORC* genes compared to the internal control *GAPDH* were determined as the value of 2^−ΔCT^.

### Standard Counting of Treg and Th17 Cells

The normal healthy controls were age-matched or relatives without detectable mutations in each allele. Briefly, 1 × 10^6^ PBMCs were surface labeled with peridinin chlorophyll protein (PerCP)-conjugated anti-CD4 (SK3, BD Biosciences, San Jose, CA) and fluorescein isothiocyanate (FITC)-conjugated anti-CD25 (2A3, BD Biosciences). The Treg cells were gated from CD4+CD25 high cells and quantified using intra-cellularly labeled phycoerythrin (PE)-conjugated anti-FOXP3 (PCH101; biosciences, San Diego, CA) (37).

Th17 cells were counted by intracellular staining of CD4+ T cells for IL-17 production after 4 h of stimulation with 50 ng/ml phorbol 12-myristate 13-acetate (PMA) and 1 μg/ml ionomycin (Sigma-Aldrich, St. Louis, MO) in the presence of 1 μl/ml GolgiStop (Becton Dickinson, Franklin Lakes, NJ) (38). After surface staining with PerCP -conjugated anti-CD4, PBMCs were fixed, permeabilized (Cytofix/Cytoperm; Pharmingen), and stained with PE-conjugated anti-IL17A (eBioscience, San Diego, LA) as previously reported (30).

Data were analyzed using CellQuest™ analysis software (BD Biosciences). The proportion of Treg cells was determined based on CD4+CD25+FOXP3+ and Th17 cells on CD4+IL17+ after non- and stimulation, respectively.

### Statistical Analyses

Data were presented as mean values from duplication in PIDs patients and healthy controls. Correlation analysis between the relative fold change of FOXP3/GAPDH or RORγt/GAPDH to Treg and Th17 cells' lymphocyte subsets was performed by regression analysis of SPSS advanced Statistical^TM^ 17 (2009, Chicago, IL). Chi-square analysis and binary logistic regression were applied to speculate whether the Treg and Th17 cell percentages were related to the phenotype of autoimmune disorders and opportunistic infections. The predicted Treg percentages, Th17 percentages and the ratio of Th17/Treg in each category were also compared by *t*-test and Mann-Whitney test in Graphpad Prism software, version 4.0 (Graphpad software Inc., San Diego, CA). A statistically significant difference was defined as *p* < 0.05.

## Results

### Linear Relationships Between the Percentages of Treg and Th17 Cells and Their Respective 2^−ΔCT(FOXP3−GAPDH)^ and 2^−ΔCT(RORγt−GAPDH)^ in Healthy Controls

**Figure 1:**
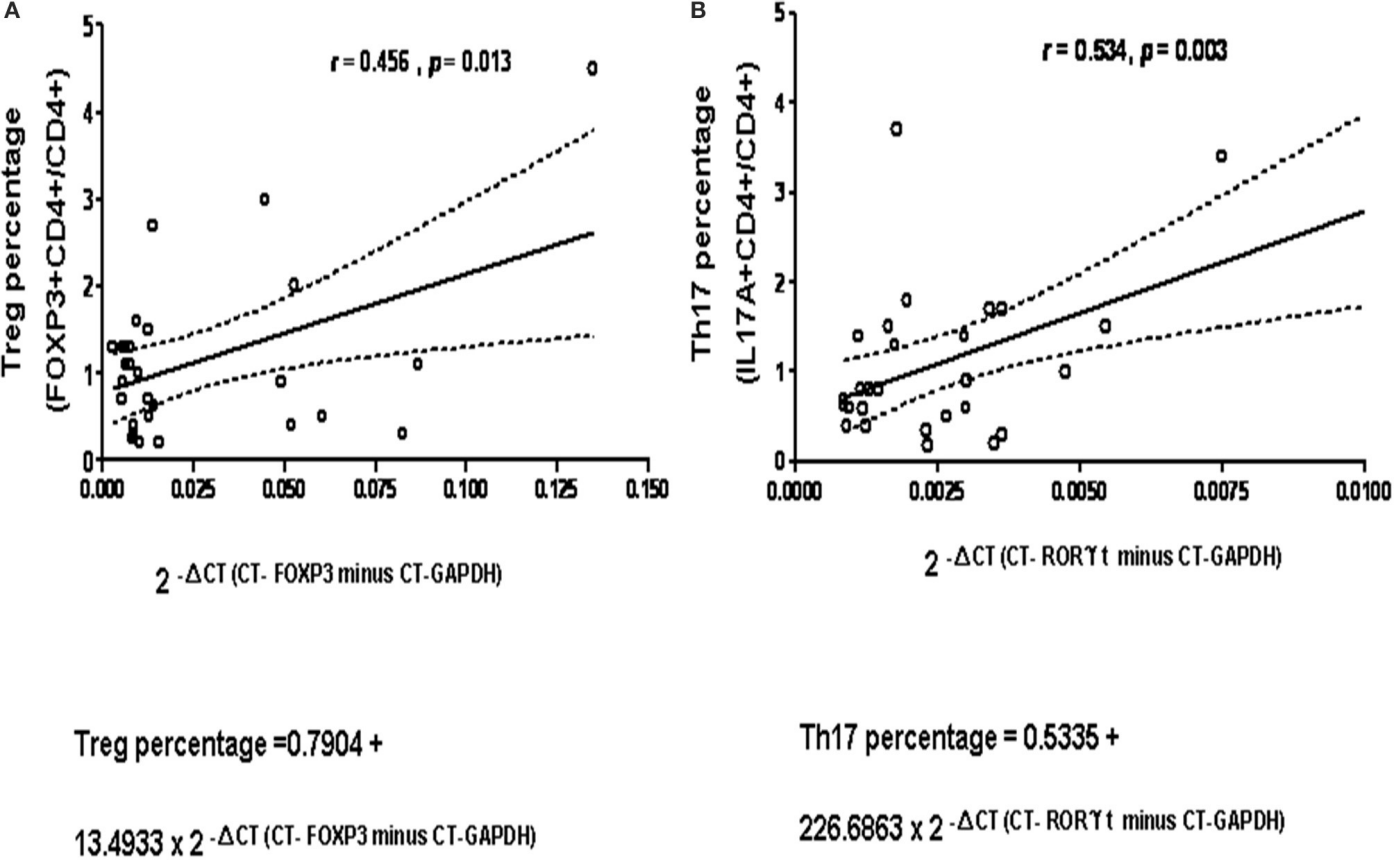
A significant positive-correction linear relationship was observed in 29 healthy controls **(A)** between the non-stimulated CD4+CD25+FOXP3 stained Treg cell subset on the X-axis and the fold expression of FOXP3/GAPDH expressed as the absolute value of 2^−ΔCT^ (as Δ CT = CT-FOXP3 minus CT-GAPDH) on the Y-axis, compatible to “Predicted Treg percentage = 0.7904 + 13.4933 × 2 ^−ΔCT(CT−FOXP3minusCT−GAPDH)^” *p* = 0.013, *R*^2^ = 0.208; and **(B)** between the stimulated CD4+IL-17A+ stained Th17 cell subset and the fold expression of RORC/GAPDH as 2^−ΔCT^ (as ΔCT = CT-RORC minus CT-GAPDH), compatible to “Predicted Th17 percentage = 0.5335 + 226.6863 × 2-^ΔCT(CT−RORγtminusCT−GAPDH)^” *p* = 0.003, *R*^2^ = 0.286. The dash curve lines meant 95% confidence interval (C.I.).

The cycle threshold (CT) values of RT-PCR for FOXP3 and RORγt compared to their own GAPDH expression were calculated in 29 age-matched controls (Supplemental Table 2). The results revealed a significant linear relationship between the non-stimulated CD4+CD25+FOXP3 stained Treg cell subset and the fold expression of FOXP3/GAPDH as the value of 2^−ΔCT=CT−FOXP3minusCT−GAPDH^ (*p* = 0.013, Figure 1A), and between the stimulated CD4+IL-17A+ stained Th17 cell subset and the fold expression of RORγt/GAPDH as the value of 2^−ΔCT=CT−RORγtminusCT−GAPDH^ (*p* = 0.003; Figure 1B).

The 5–95 percentage (%) of distribution of Treg and Th17 cells in the range transformed from the linear formula in CD4+ cells were 0.85–2.00 and 0.73–2.23%, respectively. Therefore, those below 5% were defined as “low” conditions (0.85 and 0.73%).

### The Predicted Treg Cell Percentage of the PIDs Patients Estimated From PCR-Amplified FOXP3 Expression by Positive-Correlation Linear Relationship

**Figure 2:**
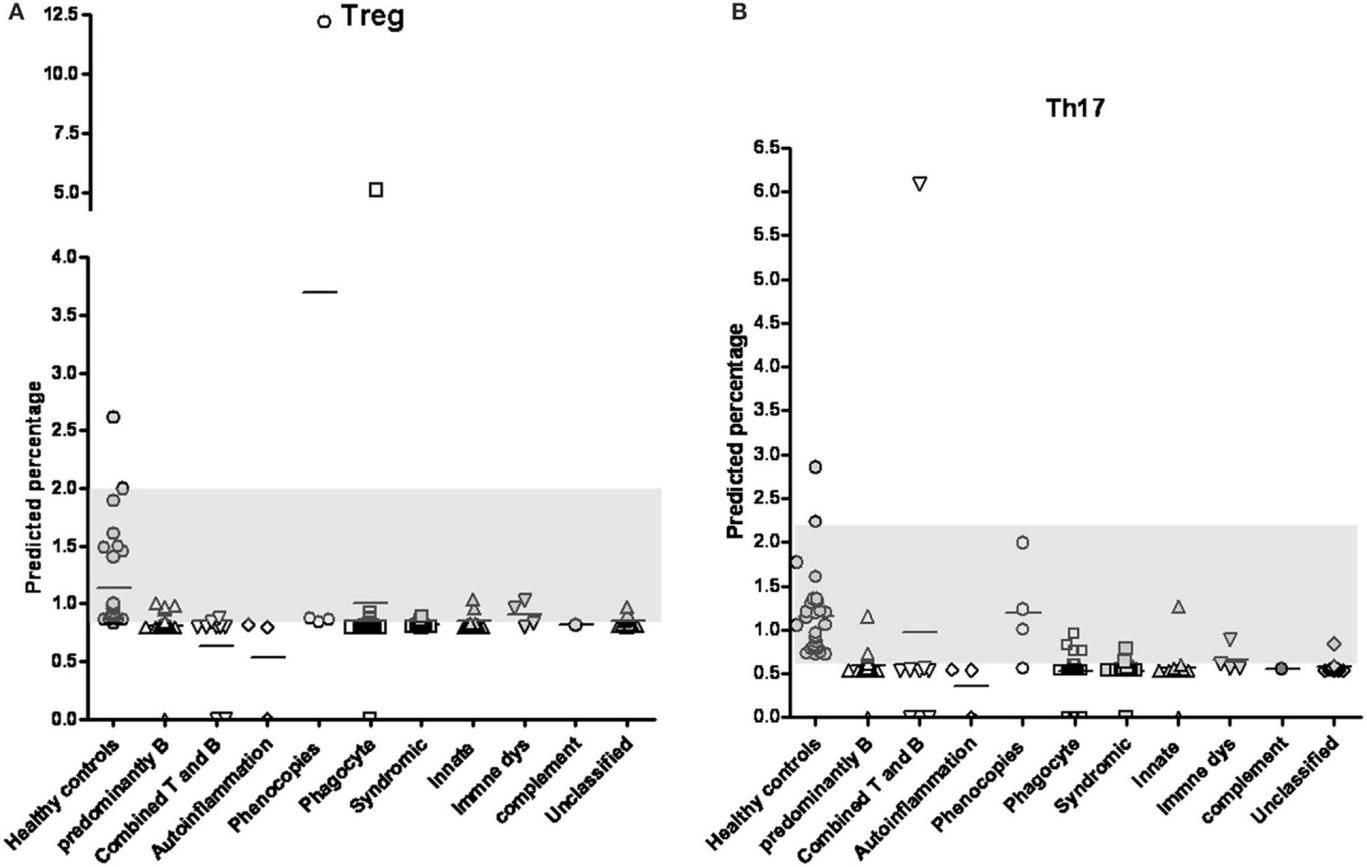
By positive-correlation linear relationship obtained from FOXP3 and RORγt expressions, the predicted percentages of Treg cells **(A)** and Th17 cells **(B)** in patients with primary immunodeficiency diseases classified into different categories were calculated. The normal distribution range of 5–95% obtained from patient-siblings or relatives with a similar age distributed as shown in the gray column (0.859–2.000% in Treg cells and 0.707–1.673% in Th17 cells; respectively). Except for the “immune dysregulation” category, the PIDs patients in the “phenocopies” category had a significantly higher Treg percentage and those in the other categories had significantly lower Treg percentages compared to the healthy controls (Supplemental Table 4). The Th17 percentages in all categories except for the “phenocopies” category were significantly lower in the PIDs patients than in the healthy controls (Supplemental Table 5).

**Figure 3:**
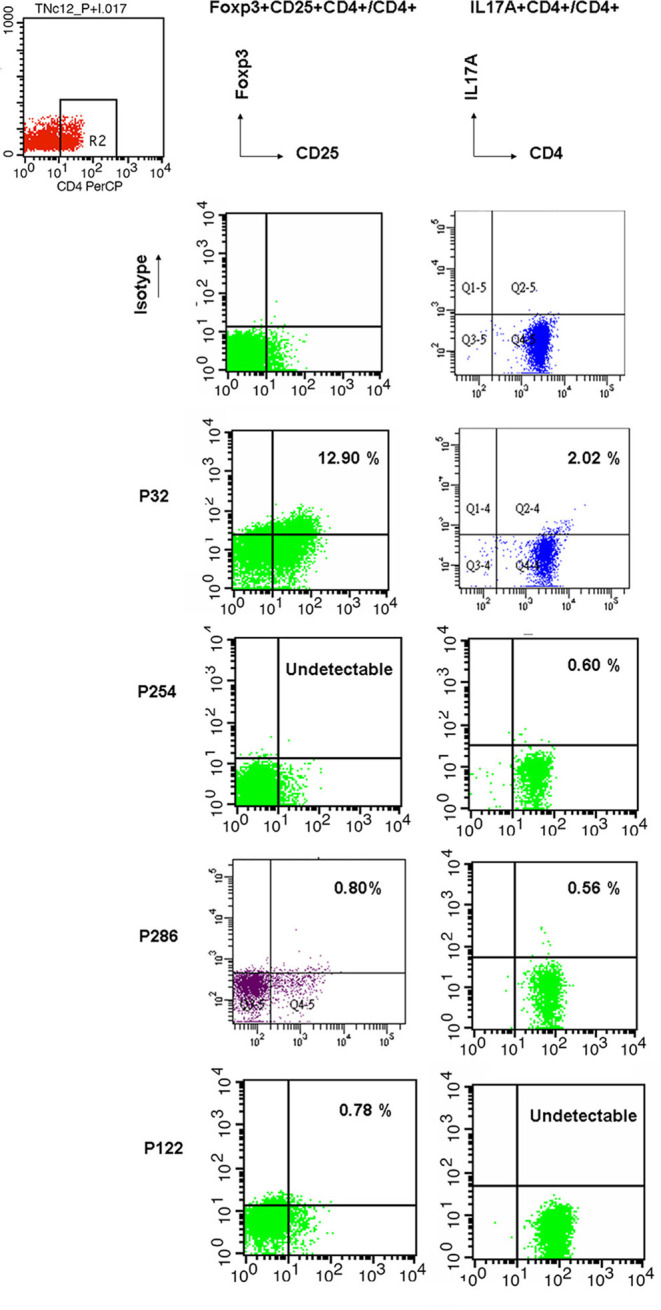
For representative flow cytometry demonstrations in PIDs patients, Treg cells, and Th17 cells were counted by intracellular PE-conjugated anti-Foxp3 without stimulation and PE-conjugated anti-IL17A staining after 4 h stimulation of 50 ng/ml PMA and 1 ug/ml ionomycin. In the right upper dot plot, higher Treg (12.90% vs. 12.216% from the linear formula) and Th17 cells (2.02% vs. 1.992% from the formula) were found in P32 patient with auto-IFNγ Abs. Undetectable Treg cell and Th17 cells were observed in P254 with the CVID phenotype and P 122 with neutropenia, respectively. Both were lower Treg (0.80% vs. 0.298% from the formula) and Th17 cells (0.56% vs. 0,537% from the formula) in P286 with the unclassified subgroup. The isotypes of antibodies to FOXP3 and Il17A were performed in the upper lane. These “true” Treg and Th17 cells were close to the predicted estimation from the fold expression of FOXP3/GAPDH and RORC/GAPDH formulae as the absolute value of 2^−ΔCT^.

Because of the low proportions of Treg and Th17 cells and the limited availability of peripheral blood from patients with PIDs, residual complement DNA was used to estimate the percentages of lymphocytes using a linear relationship: The predicted Treg percentage was calculated as 2^−ΔCT=CT−FOXP3minusCT−GAPDH^ multiplied by 13.4933 + 0.7904 (Figure 1A and a simple representation of RT-PCR is shown in Supplemental Figure 1). Among 91 patients in the updated PIDs categories (Supplemental Table 3), 68 with a decreased predicted percentage of Treg cells met the definition of “low” threshold (Figure 2A and Supplemental Table 4). Of these patients, 15 (of 19) had predominant antibody deficiencies, eight (of nine) had combined deficiencies, three (all) had auto-inflammatory disorders, 14 (of 19) had congenital defects of phagocytes, 11 (of 12) had combined immunodeficiencies with associated or syndromic features, seven (of nine) had defects in innate immunity, two (of four) including one XIAP mutation (P296) had immune dysregulation diseases, and six (of 11) including one with alopecia and albinism (P286) were unclassified, as demonstrated by flow cytometry (Figure 3; their predicted percentages are shown in Supplemental Table 3). Among them, five patients with CVID (P254), periodic fever (P238), and STAT1 (P54) each and two with SCID (P394 and P365) had undetectable FOXP3 production and profound Treg cell deficiency.

Compared to the normal controls, all categories had significantly lower Treg cell percentages (*t*-test and Mann-Whitney test, *p* < 0.05) except for the “Phenocopies of PID” category which had a significantly higher percentage and the “Disease of immune dysregulation” category which had a similar percentage (Figure 2A and Supplemental Table 4). Those with more homozygous diseases of BTK mutations, CVID, CID (SCID), WAS, and CGD had consistent comparisons (Supplemental Figure 2).

### The Predicted Th17 Cell Percentage of the PIDs Patients Estimated From PCR-Amplified RORγt Expression by Positive-Correlation Linear Relationship

Based on the formula of Th17 percentage as 2^−ΔCT=CT−RORγtminusCT−GAPDH)^ multiplied by 226.6863 + 0.5335 (Figure 1B), only 13 patients had a normal predicted percentage of Th17 cells, including neutropenia (P98), WAS (P247), IFNGR (P132), NEMO (P369), HLH (P214), and refractory TB (P96) each and two CVID (P27 and P164), three auto-IFN-gamma (P32, P188 and P197) and two gp91 (P22 and P227). Almost all PIDs patients had a relatively low level of predicted Th17 cells (Figure 2B and Supplemental Table 5) except for those in the “phenocopies of PID” category who had a similar Th17 percentage to the controls. Comparisons between the patients with specific monogenetic PIDs, including BTK mutations, CVID, CID (SCID), WAS, and CGD were also consistent (Supplemental Figure 3).

Ten patients had “undetectable” RORγt, including those with CVID (P433), periodic fever (P238), STAT1 (P54), WAS (P154), chronic muco-cutaneous candidiasis (P379), two with neutropenia (P63 and P66), and three with SCID (P140, P394, and P365). Neither anti-IL-17 autoantibodies nor RORγt mutations were identified in these patients. Of these patients, four (P54, P238, P365, and P394) also had an undetectable FOXP3 expression.

### Correlation Analysis of Th17 Cell Proportions, Treg Cell Proportions and Th17/Treg Ratio to Tested Ages

**Figure 4:**
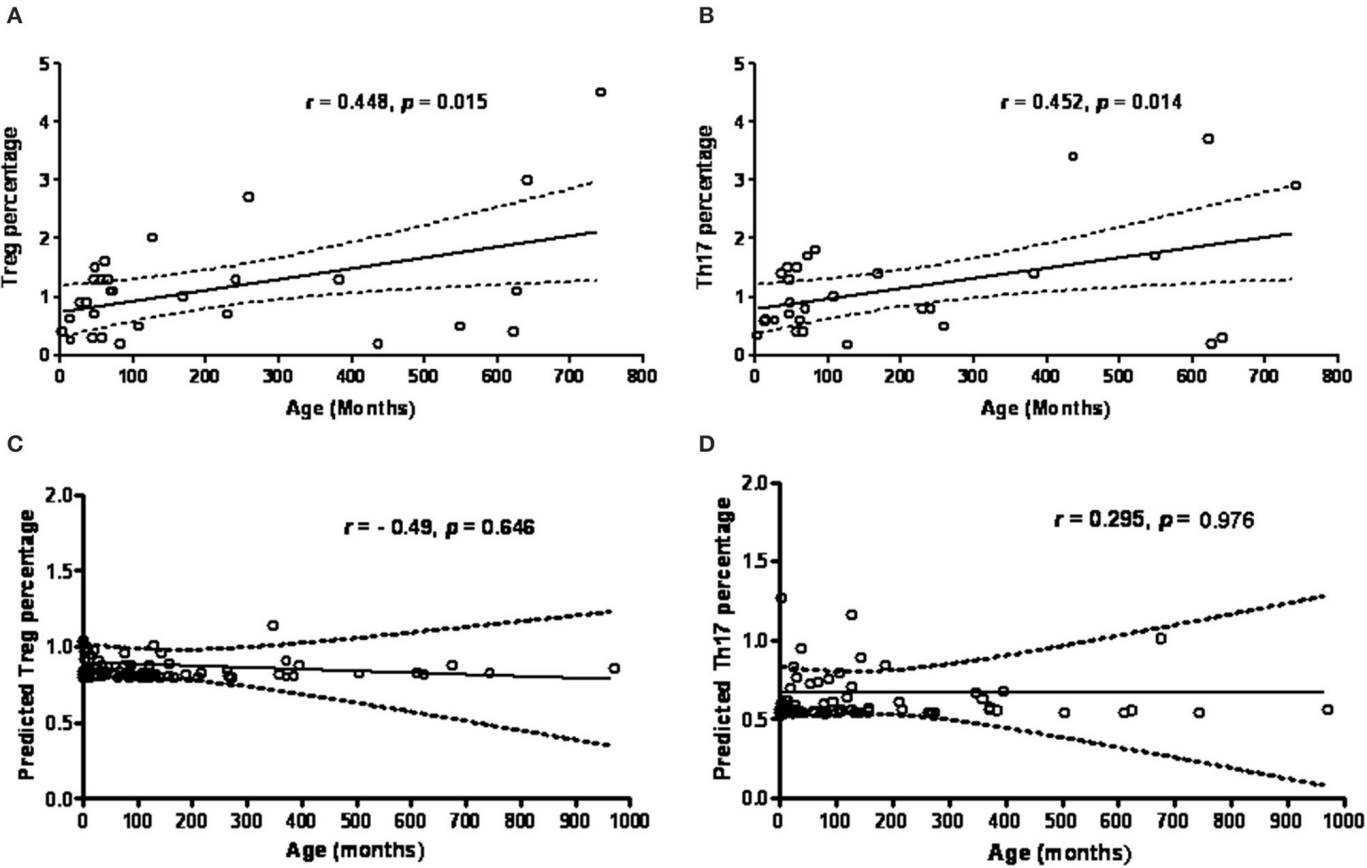
The age (months) factor is in positive proportion to the percentage of Treg cells **(A)** and Th17 cells **(B)** by intracellular staining in healthy controls. However, the predicted Treg cells and Th17 cells estimated from RT-PCR amplified FOXP3 **(C)** and RORγt expressions **(D)** in PIDs patients seemed to be more independent of the age factor. Statistics was executed by Pearson correlation and *p* < 0.05 was significant.

There was a positive correlation/linear relationship trend between age (months) and percentage of Treg cells (*r* = 0.448, *p* = 0.015) and percentage of Th17 cells (*r* = 0.452, *p* = 0.014) in the healthy controls (Figures 4A,B). In contrast to the controls, age in the PIDs patients was not significantly associated with the calculated Treg and Th17 percentages (*p* > 0.05, Figures 4C,D).

**Figure 5:**
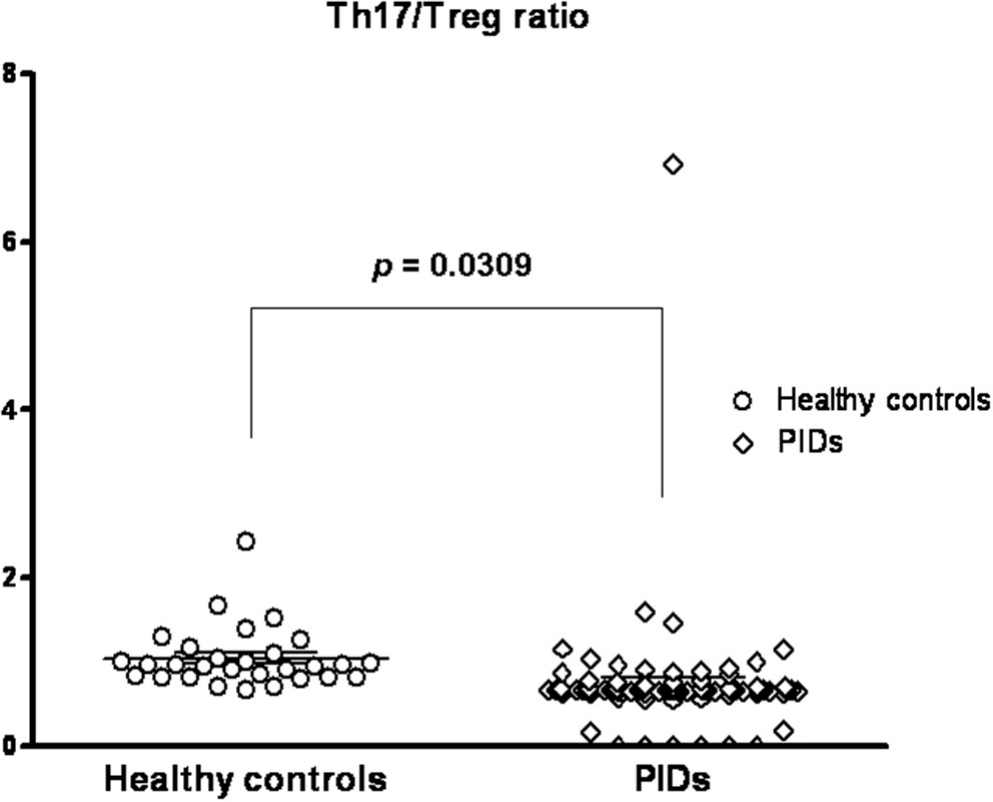
Compared to the healthy controls (mean ± SEM; 1.044 ± 0.6688), the PIDs patients had a significantly lower predicted Th17/Treg ratio (0.7378 ± 0.07807; *p* = 0.0309).

Thus, the healthy controls had a trend of an age-dependent Th17 cell subpopulation [range 0.7–2.9% by intra-staining of IL-17A as shown in Figure 4B, as previously reported (8)]. The PIDs patients had relatively lower calculated Th17 and Treg percentages independent of age. In addition, the PIDs patients with a greater decrease in Th17 cells and relatively lower decrease in Treg cells had a significantly lower Th17/Treg ratio (mean ± SEM; 0.7378 ± 0.07807 vs. 1.044 ± 0.6688; *p* = 0.031) (Figure 5).

### The Predicted Treg and Th17 Percentages on the Phenotypes of Autoimmune Disorders/Opportunistic Infections

There were 51 episodes of autoimmune disorders in 42 patients and 46 episodes of opportunistic infections in 33 patients, except for four patients (autoinflammation in three and complement 3 deficiency in one) without these manifestations (Table 1). The patients with “combined T and B deficiency” and “phagocytic disorders” often presented with more than one opportunistic infection, including candidiasis, Bacillus Calmette-Guerin-related, pneumonitis, NTM and aspergillosis. The patients with “combined immunodeficiency and syndromic features” and “innate immunity” had a relatively higher incidence of autoimmune disorders, notably severe atopic dermatitis (erythroderma-like), herpes-like dermatitis, lymphadenopathy, hepatosplenomegaly, refractory IBD-like diarrhea and lymphoma.

**Table 1:** Episodes of opportunistic infections and autoimmune disorders in the affected PIDs patients in separate categories.

**Symptoms/Signs**	**Total**	**Predominantly B**	**Combined**	**Phenocopies**	**Phagocyte**	**Syndromic**	**Innate**	**Immune Dys**.	**Unclassified**
Subgroup patients Affected numbers^*^	**87**	**19**	**9**	**4**	**19**	**12**	**9**	**4**	**11**
**OPPORTUNISTIC INFECTIONS**									
Candidiasis	12		7		2		1	2	
BCG-related infection	10		1		9				
Pneumonitis (subgroup)	8								
*Pneumocystis jirovecii* pneumonitis	(5)		5						
*Cytomegalovirus*	(1)		1						
Adenovirus	(1)	1							
Unknown	(1)		1						
Non-tuberculosis mycobacterium	7			3					4
Aspergillosis	6				6				
Tuberculosis mycobacterium	1								1
Varicella	1			1					
*Talaromyces* (Penicillium) marneffei	1			1					
**AUTOIMMUNE DISORDERS**									
Severe atopic dermatitis	8					7			1
Lymphadenopathy	8	1	1	3	3				
Herpes-like dermatitis	7						7		
Hepatosplenomegaly	6	1	4		1				
Refractory diarrhea (IBD-like)	4	1				1		2	
Hemophagocytic lymphohistiocytosis	3							3	
Lymphoma	3					2			1
Granuloma (subgroup)	3								
Lung	(2)				2				
Skin	(1)					1			
Idiopathic thrombocytic purpura	2					2			
Alopecia	2						1		1
Splenomegaly	1				1				
Takayasu's vasculitis	1	1							
Liver cirrhosis	1	1							
Hypothyroidism	1				1				
Albinism	1								1

* *The subgroup with autoinflammation (3 patients) and complement (1 patent) diseases were free of opportunistic infections and autoimmune disorders*.

**Figure 6:**
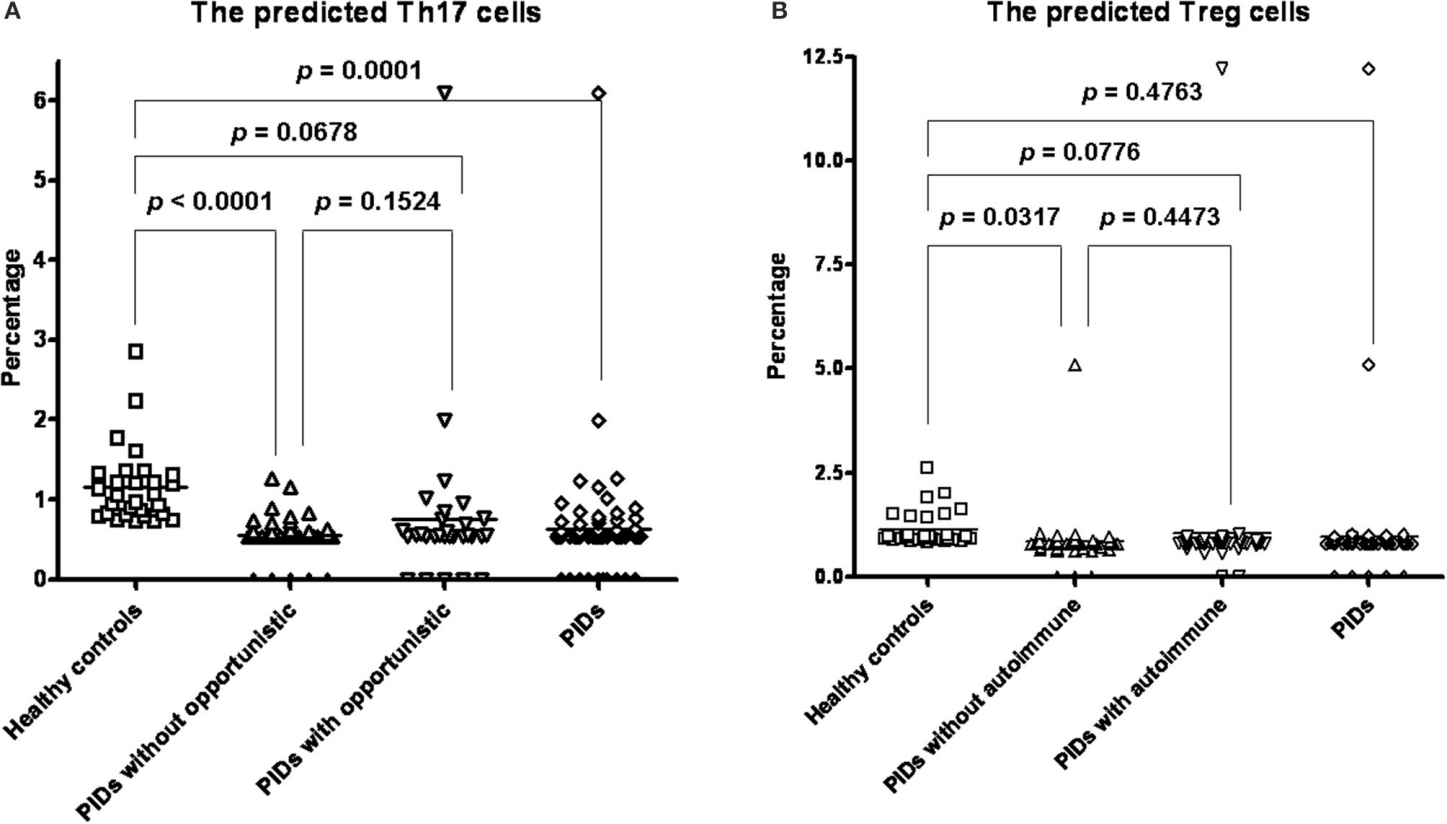
Compared to the healthy controls (mean ± SEM; 1.159 ± 0.1674%), all PIDs (0.6242 ± 0.06851%, *p* = 0.0001) and those without opportunistic infections (0.5512 ± 0.02884%; *p* < 0.0001) had a significantly lower predicted Th17 percentage. There was no significance between those with (0.7565 ± 0.1851; *p* = 0.1524) and without opportunistic infections **(A)**. PIDs patients without autoimmune disorders had a significantly lower Treg cell percentage (0.8362 ± 0.09561%, *p* = 0.0317) than the healthy controls (1.139 ± 0.08049%). There was no significant difference between all PIDs patients (0.9641 ± 0.1333%) and the healthy controls (*p* = 0.4763); and between those with (1.044 ± 0.2676%, *p* = 0.4473) and without autoimmune **(B)**.

The predicted Th17/Treg ratio in every category except the “autoinflammation” and “phenocopies” categories was significantly lower than that in the controls (Supplemental Table 6). When we focused on more specific diseases, including BTK, CVID, SCID and CID, CGD and WAS, we also found significantly lower predicted Th17/Treg ratios than in the controls (Supplemental Table 7). We further sub-grouped those with or without the phenotype of autoimmune/opportunistic infections in all PIDs, by breaking the category-barrier, and investigated whether the calculated Treg percentage was correlated to the autoimmune phenotype and the calculated Th17 percentage to opportunistic infections. The predicted Th17 percentages in all PIDs patients (0.6242 ± 0.06851%, *p* = 0.0001) and those without opportunistic infections (0.5512 ± 0.02884%; *p* < 0.0001) were significantly lower than those in the healthy controls (1.153 ± 0.08893%) (Figure 6A). The predicted Treg percentage in the PIDs patients without autoimmune disorders was significantly lower than that in the healthy controls (1.139 ± 0.08049% and 0.8362 ± 0.09561%, *p* = 0.0317; Figure 6B). In other subgroups of low/non-low predicted Treg, the Th17 percentages and Th17/Treg ratio were not statistically significant by chi-square analysis and binary logistic regression (Supplemental Tables 8, 9) for the development of the autoimmune phenotype and opportunistic infections. There were no significant differences between the PIDs patients with/without opportunistic infections and those with/without autoimmune disorders, which suggested that other factors beyond Th17 and Treg cells could contribute to opportunistic infections/autoimmune disorders.

## Discussion

Lower Treg and Th17 cell percentages predicted by RT-PCR-amplified FOXP3 and RORγt below 5% of the normal range were common in 68 and 78 of the 91 PIDs patients. FOXP3 was undetectable in one patient (CVID), and RORγt was undetectable in six patients (one CVID, one CID, two neutropenia, one WAS, and one CMC). Both FOXP3 and RORγt were undetectable in three patients with IL2RG, RAG1 and STAT1 mutations, implying that Treg and Th17 cells were almost completely absent, and therefore they had increased risks of developing autoimmune disorders and opportunistic infections. Early HSCT rescued our patient with a RAG1 mutation (P365) and stopped the development of an autoimmune disorder (Omenn syndrome). In Taiwan, aggressive interventions through the *National Health Insurance* and *Rare Disease Control and Orphan Drug Act* for newborn TREC screening including regular immunoglobulin infusions, adequate prophylactics and transplantation in the early stage of the initial disease course have prevented the development of autoimmune disorders and opportunistic infections, and this might explain comparison bias in PIDs patients between the Treg/Th17 cellular proportions at diagnosis and the development of autoimmune disorders/opportunistic infections.

Our patients with *IL2RG, RAG1/2*, and *AICDA* mutations had Treg cell dysfunction causing lymphadenopathy and erythroderma (or severe atopic dermatitis) similar to Omenn syndrome, which is consistent with previous studies (39–42). Inflammatory bowel disease and endocrine anomalies were frequently observed in our patients with CVID, *STAT1* mutations, and auto-inflammation (43–48). Defective IL12/23 signaling (caused by mutations of the *IL-12p40, STAT3*, and *STAT1* genes) and IL17 feedback (due to mutations of the *IL17A, IL17F*, and *RORC* genes, but not yet identified in our patients) impair Th17 cell development (45, 49, 50), thereby inhibiting the IL-17 family of cytokines for pro-inflammatory chemokines, antimicrobial peptides, and matrix metalloproteinases against extracellular bacteria and fungi (51). Relatively decreased expressions of RT-PCR-amplified FOXP3 and/or RORγt were noted in the “predominantly antibody deficiencies” (Btk, CVID and TTC37), “combined T and B immunodeficiencies” (SCID and CID), “congenital phagocyte disorders” (CGD), “combined immunodeficiencies with associated or syndromic features” (Wiskott-Aldrich syndrome), and “disease of immune dysregulation” (XIAP) categories, and in the “unclassified” category encompassing patients with alopecia, albinism, severe atopic dermatitis or non-tuberculosis infections. This again highlights that Treg and Th17 cell deficiencies often involve many categories of PIDs.

Direct staining and linear relationship analysis showed that the highest Treg percentage was in patients with a large volume of pathologic auto-interferon γ antibodies. Such patients may have physiologically enriched Treg cell pools to “suppress” these neutralizing autoantibodies that interrupt the IL12/23-interferon γ (IFNγ) circuit rather than Th17 deficiency. This could then increase susceptibility to intracellular mycobacterial and salmonella infections, because the other three patients with the highest mean Th17 percentage (1.20%) in the “phenocopies” category had a large disproportional volume of auto-interferon γ antibodies blocking IFNγ signaling for Th1 shift and thus skewing into an elevated percentage of Th17 cells. Alternatively, the lowest Treg percentage and even undetectable *FOXP3* gene expressions were found in the “autoinflammation” category. This suggests that insufficient Treg cells were not able to overcome the boosting self-inflammation process, thereby resulting in periodic fever.

In contrast to the healthy controls who showed a trend of age-dependent Treg cell subpopulations (Figure 4A) (52, 53), the majority of the studied PIDs patients had a more age-independent predicted Treg cell subpopulation (Figure 4C), but over 85% (78/91) maintained relatively lower Th17 populations that inhibited human B cell survival and activation of antibody-secreting plasma cells (54). Th17 cell deficiency can, in part, elucidate the mechanism of hypogammaglobulinemia in “predominately antibody deficiencies,” “combined T and B deficiencies,” and “associated or syndromatic features (WAS, STAT3, and ATM).” This is why the predicted Th17/Treg ratio in the PIDs patients was opposite to the that in the patients with SLE (55), vasculitis (56), and exacerbated asthma (57) who had elevated levels of Th17 cells and decreased levels of Treg cells.

The study should be interpreted in light of its limitations. First, cross-sectional assessments of FOXP3 and RORγt expressions at diagnosis may have been affected by subclinical infections with Th1 deviation. Longitudinal follow-up studies of Treg and Th17 populations should provide more real correlations to the whole phenotypic spectra. Second, further studies should investigate cell-contact Treg suppression and functional cytokine production (IL-10, TGF-β, and IL17) if more blood samples are available, because isolated lymphocytes of CGD patients have been shown to secrete higher levels of IL17A *in vitro* in co-cultures with CD3CD28 (58). Third, the predicted Treg and Th17 percentages were calculated from their RT-PCR-amplified FOXP3 and RORC expressions rather than from direct counting and even four-color for Treg staining (CD4+ CD25hi CD127lowFoxp3+) because of FOXP3 not exclusively expressed by Treg cells, although equal values were obtained from the formula prediction and current staining (CD4+CD25+FOXP3+ for Treg and CD4+IL17+ for Th17) in selected patients. Fourth, diminished T follicular help (Tfh) cells can attenuate vaccine response and decrease the development of Th17 cells. Further studies are warranted to elucidate the mechanisms of Th17 cell impairment, including at least Tfh cells.

In conclusion, a decrease in the transcription factor RORγt for Th17 cell development in the PIDs patients increased susceptibility to (opportunistic) infections. An age-independent but relatively lower FOXP3 expression for Treg cell development was associated with autoimmune disorders despite a reversed Th17/Treg ratio in contrast to that in the patients without PIDs. These findings override the different updated categories of PIDs and could explain the potential phenotype of infections and autoimmunity, although early interventions have prevented such phenotypic appearances and may have introduced statistical bias. The RORγt and FOXP3 transcription factors may serve as biomarkers to establish threshold values for infection and autoimmunity and allow for individualized optimal prophylactic and immune reconstruction interventions.

## Data Availability Statement

The raw data supporting the conclusions of this article will be made available by the authors, without undue reservation, to any qualified researcher.

## Ethics Statement

The Chang Gung Human Investigation Committee approved this study. Written informed consent was obtained from the individual(s), parents, and legal guardians for the publication of any potentially identifiable images or data included in this article.

## Author Contributions

W-IL carried out the molecular genetic studies, participated in the sequence alignment, and drafted the manuscript. S-JL and L-SO carried out the immunoassays. W-IL and Y-FS participated in the sequence alignment. W-IL and J-LH designed the study and performed the statistical analysis. K-WY, L-CC, T-CY, C-YW, and T-HJ cared critical patients. J-LH conceived the study, participated in its design and coordination, and helped in drafting the manuscript. All authors read and approved the final manuscript.

## Conflict of Interest

The authors declare that the research was conducted in the absence of any commercial or financial relationships that could be construed as a potential conflict of interest.

## Abbreviations

AICDA: activation induced cytidine deaminase
AIRE: AutoImmune REgulator
APECED: Autoimmune PolyEndocrinopathy with Candidiasis and Ectodermal Dystrophy
BCG: Bacillus Calmette-Guerin
Btk: Bruton's tyrosine kinase
CGD: chronic granulomatous disease
CID: combined T and B immunodeficiency
CMC: chronic mucocutaneous candidiasis
CVID: common variable immunodeficiency disease
DOCK8: dedicator of cytokinesis 8
FOXP3: forkhead box P3
GAPDH: glyceraldehyde 3-phosphate dehydrogenase
HIES: Hyper-IgE recurrent infection syndromes
HLH: hemophagocytic lymphohistocytosis
IBD: inflammatory bowel disease
NEMO: Nuclear factor-kappa B Essential Modulator
NTM: non-tuberculosis mycobacteria
PBMCs: peripheral blood mononuclear cells
PMA: phorbol 12-myristate 13-acetate
RORγt: retinoic acid receptor-related orphan nuclear receptor gamma T
RT-PCR: real time polymerase chain reaction
STAT3: signal transducer and activator of transcription 3
TYK2: tyrosine kinase 2
TTC37: tetratricopeptide repeat domain 37
WAS: Wiskott-Aldrich syndrome
XIAP: X-linked inhibitor of apoptosis.
