# Supplementary material for: Lower T Regulatory and Th17 Cell Populations Predicted by RT-PCR-Amplified FOXP3 and RORγt Genes Are Not Rare in Patients With Primary Immunodeficiency Diseases
Source: Front Immunol. 2020 Jun 25;11:1111. doi: 10.3389/fimmu.2020.01111 (PMC7330141; doi:10.3389/fimmu.2020.01111)
Supplement: Supplementary file 1 [file Data_Sheet_1.pdf]

Supplemental Table 1. Distribution patterns in 250 patients with PIDs (219 unrelated families)

Disease category	Reported		Mortality	
	F	M	F	M
**Predominate antibodies deficiencies**	**19**	**38**	**0**	**2**
Common variable immunodeficiency (CVID)	12	16		
Agammaglobulinemia	1	2		
X-linked agammaglobulinemia (XLA)	0	12		2
Selective immunoglobulin deficiency [a]	6	3		
Transient hypogammaglobulinemia of infant (THI)	0	5		
**Combined immunodeficiencies**	**9**	**31**	**4**	**10**
Severe combined B and T-cell Immunodeficiency (SCID)	7	15	4	8
Omenn syndrome	1	1		
Hyper IgM syndrome (HIGM)	0	13		1
Combined immunodeficiencies with predominant T-cell defect [b]	1	1		1
(MHC Class I deficiency) [c]	0	1		
**Congenital defects of phagocyte number, function, or both**	**8**	**27**	**1**	**5**
Chronic granulomatous disease (CGD)	2	19		3
Chronic severe neutropenia	4	2		
Interferon-γ associated immunodeficiency [d]	1	3		
Leukocyte adhesion disease (LAD)	1	1	1	1
Bater syndrome	0	1		
Nature killer cell deficiency [f]	0	1		1
**Combined immunodeficiencies with associated or syndromic features**	**28**	**61**	**3**	**11**
DiGeorge syndrome (DGS)	16	25	1	2
Wiskott-Aldrich syndrome (WAS)	1 [e]	21		6
Hyper IgE syndrome (HIES)	7	4	1	2
Ataxia telangiectasis (DNA breakage associated syndrome; AT)	2	4	1	1
Comel-Netherton syndrome	2	4		
Cartilage-hair hypoplasia	0	1		
Chronic mucocutaneous candidiasis	0	1		
Primary CD4 T cell deficiency [f]	0	1		
**Disease of immune dysregulation**	**2**	**4**	**2**	**2**
Chediak-Higashi syndrome (CHS)	1	1	1	
Familial hereditary hemophagocytosis	1	0	1	
X-linked lymphoproliferative syndrome	0	1		
Immunodeficiency polyendocrinopathy enteropathy X-linked	0	2		2
**Defects in innate immunity**	**0**	**2**		**2**
Anhidrotic ectodermal dysplasia with immunodeficien	0	2		2
**Auto-inflammatory disorders**	**1**	**1**		
Cold-induced auto-inflammatory disease (CIAS)	1	1		
**Complement deficiencies**	**6**	**9**		**1**
Primary C3 deficiency	0	1		
Primary C7 deficiency	1	1		
Hereditary angioedema	5	7		
**Phenocopies of PIDs**	2	2		
Auto-interferon-gamma antibodies	2	2		
**Total**	**75**	**175**	**10**	**33**

Abbreviations: F, female; M, male

[a]Selective immunoglobulin deficiency includes IgA (1 female), IgG2 sub-class (2 females, 1 male), IgG2 and IgG4 sub-classes (1 female, 1 male), IgG3 sub-class (1 male), and IgG3 and IgG4 sub-classes (2 females) deficiencies. No patients had selective deficiency to polysaccharide.

[b]Commonly referred to as Nezelof syndrome.

[c]Patient had decreased expression of MHC class I compared to normal controls, but normal MHC class II. His family refused further genetic analysis.

[d]*Interferon-γ associated immunodeficiency* contains patients with mutations of IL-12RB1, IFNGR1, IFNGR2, IL-12p40, STAT1, or NEMO deficiency.

[e]The female Wiskott-Aldrich syndrome variant was previously reported (Lin and Hsu, 1984).

[f]These two diagnosis of *Nature killer cell deficiency* and *Primary CD4 T cell deficiency* were beyond the update eight categories (Tangye SG, et al. 2020).[31] By lymphocyte subsets and clinical presentations, we classify *Nature killer cell deficiency* into the phagocyte defects and *Primary CD4 T cell deficiency* with normal immunoglobulin levels and without recurrent sino-pulmonary infections into “combined immunodeficiencies with associated or syndromic features”.

Supplemental Table 2. Flow cytometric staining of intracellular FOXP3 and IL17A+ in peripheral blood mononuclear CD4+ cells with and without stimulation with phorbol 12-myristate 13-acetate (PMA) and ionomycin and the cycle threshold (CT) of real-time polymerase chain reaction (RT-PCR) amplification for RORγt, FOXP3, and GAPDH isolated from peripheral blood mononuclear cells from the healthy controls

Controls		IL17A+CD4+ /CD4+ (%)		Foxp3+CD25+CD4+ /CD4+ (%)		CT-RT-PCR				
	Age (M)	Non-stimulated	Stimulated	Non-stimulated	Stimulated	RORγt	FOXP3	GAPDH	RORγt-GAPDH	FOXP3-GAPDH
N1	12	0.0	0.6	0.6	3.2	31.95	28.38	22.21	9.74	6.17
N2	14	0.0	0.6	0.3	0.7	33.12	29.88	22.94	10.22	6.98
N3	8	0.2	0.3	0.4	1.1	32.78	28.28	24.01	8.77	4.27
N4	127	0.1	0.2	2.0	1.4	31.58	27.08	22.83	8.75	4.25
N5	45	0.0	0.8	0.3	1.2	32.02	28.12	24.50	7.52	3.62
N6	627	0.6	1.5	1.1	1.2	33.12	28.47	24.94	8.16	3.53
N7	549	0.2	0.2	0.5	1.2	31.94	27.79	23.74	8.20	4.05
N8	742	0.4	0.3	4.5	5.3	31.19	27.48	24.59	6.61	2.89
N9	642	0.6	0.5	3.1	2.6	31.34	27.69	23.23	8.11	4.46
N10	259	0.5	0.6	2.7	1.4	31.22	28.86	22.66	8.56	6.21
N11	27	0.0	0.4	0.9	0.3	31.78	27.74	23.39	8.39	4.35
N12	56	0.0	1.7	1.3	0.2	32.25	31.18	22.59	9.66	8.59
N13	241	0.1	2.9	1.3	2.4	33.21	30.92	23.62	9.61	7.32
N14	169	0.2	1.4	1.2	2.2	34.27	32.56	25.87	8.42	6.69
N15	230	0.0	0.8	0.7	1.4	33.64	31.81	24.21	9.44	7.61
N16	383	0.1	1.4	1.3	2.1	34.44	32.17	24.62	9.84	7.57
N17	623	0.4	3.7	0.4	1.0	34.37	32.15	25.23	9.14	6.92
N18	36	0.5	1.4	0.9	0.4	32.42	31.60	24.02	8.41	7.58
N19	107	0.0	1.2	0.5	0.7	32.32	30.91	24.61	7.72	6.30
N20	46	0.7	1.3	0.7	0.7	33.36	30.53	24.19	9.17	6.34
N21	57	0.9	1.5	0.3	0.9	34.45	32.07	25.18	9.27	6.89
N22	437	0.3	3.4	0.2	0.3	32.12	31.81	25.06	7.06	6.75
N23	72	0.2	1.7	1.1	1.4	31.94	30.93	23.83	8.11	7.12
N24	82	0.6	1.8	0.2	2.2	35.78	32.82	26.78	9.04	6.02
N25	69	0.1	0.8	1.1	2.5	34.38	31.90	24.63	9.78	7.30
N26	48	0.1	0.9	1.5	1.5	33.19	31.15	24.81	8.38	6.34
N27	66	0.2	0.4	1.3	1.2	34.18	31.59	24.04	10.14	7.55
N28	62	0.0	0.6	1.6	3.4	35.47	32.17	25.40	10.07	6.77
N29	47	0.1	0.7	1.3	2.6	33.22	30.06	23.21	10.22	7.06

Supplemental Table 3. Tested age, genetic mutations, predicted Th17 and Treg percentages from lineal regression of $2^{-\Delta CT}$ and the ratio in PIDs patients

Patient / sex	Tested age (Months)	Mutations or/ clinical diagnosis	GAPDH CT value	△CT RORγt-GAPDH	Predicted Th17 percentage (0734379 2.232322)	△CT FOXP3-GAPDH	Predicted Treg percentage (0.859495-1.995686)	Predicted Th17/Treg ratio (0.706629-1.672747)	Clinical presentation	
									Opportunistic infection	Autoimmune/Inflammation disorder
**Predominantly antibody deficiencies**										
P4 / M	40	Btk Int 14 (-2)A>G; skip exon 14	21.64	14.72	0.541878	8.10	0.839605	0.642857		
P5 / M	14	Btk Int 14 (-2)A>G; skip exon 14	24.03	11.40	0.617364	6.41	0.949104	0.652632		
P138 / M	9	Btk n.1821C>T; p. Arg 641 Cys	19.61	18.28	0.534191	13.73	0.791419	0.670886		
P223 / M	29	Btk n.36G>T, p. Lys12Asn	20.53	14.87	0.541049	10.03	0.803332	0.673506		
P159 / M	110	Btk n.232C>T, p. Glu78Stop	24.59	13.29	0.556111	9.05	0.815883	0.682927		
P200 /M	36	Btk n. 1042T>G, p. Phe304Val	21.07	15.47	0.538473	10.45	0.800072	0.673031		
P52 / M	273	Undefined/CVID	21.03	14.65	0.542296	10.72	0.798426	0.679207		Hepatosplenomegaly
P71 / F	744	Undefined/CVID	17.98	14.65	0.542296	8.76	0.82155	0.658537		Lymphadenopathy
P11 / M	129	Undefined/CVID	20.76	10.38	0.703590	10.07	0.802979	0.876225		Takayashu' vasculitis
P164 / M	129	Undefined/CVID	32.90	8.51	1.155292	5.97	1.005689	1.148515		
P254 / M	127	Undefined/CVID	27.85	12.96	0.561928	undetectable	undetectable			
P433 / F	167	Undefined/CVID	26.06	undetectable	undetectable	13.77	0.791392	zero		
P55 / M	85	Del chr 1.42 / HIGM	17.41	17.24	0.534943	8.27	0.834138	0.638554		
P323 / F	20	AICDA, Del 37Asp38Ser	20.73	10.44	0.696661	6.17	0.977823	0.714286	Adenovirus pneumonitis	
P285 / M	612	Thymoma	18.94	14.79	0.541481	8.77	0.821335	0.658537		
P429 / F	5	TTC37, 3724 ins A, Tyr1169Ter/refractory diarrhea	22.58	13.46	0.553600	6.25	0.967715	0.572065		Liver cirrhosis, refractory diarrhea
P350 / M	2	Undefined/THI	21.02	16.16	0.536575	9.16	0.814014	0.666667		
P155 / F	54	Undefined/CIVD	30.18	10.25	0.719631	8.88	0.819066	0.878049		
P27 / M	67	Undefined/CIVD	28.17	10.13	0.735777	8.26	0.834442	0.891566		

**Combined T and B immunodeficiencies**											
P9 / M	10		IL2RG, Trp74Gly	19.98	13.15	0.558418	10.76	0.798207	0.699590	Oral candidiasis, PJP	Hepatosplenomegaly, erythroderma
P140 / M	6		IL2RG, Arg226Cys	24.27	undetectable	undetectable	14.90	0.790868	zero	Oral candidiasis, PJP	
P345B / M	1		Undefined/SCID	19.50	18.03	0.534326	14.55	0.790989	0.670886	Oral candidiasis, PJP	
P394 / M	3		IL2RG, n.854G>A, skip exon 6	27.53	undetectable	undetectable	undetectable	undetectable	D	Oral candidiasis, PJP	Hepatosplenomegaly
P345 / M	2		Undefined / SCID	17.54	20.86	0.533598	17.71	0.790489	0.670886	Oral candidiasis, PJP	
P365 / F	4		RAG1, Leu474Arg, Arg776Gln	30.11	undetectable	undetectable	undetectable	undetectable	D	Pneumonitis	
P325 /M	1		Undefined/CID	19.09	17.81	0.534465	11.84	0.794107	0.670886	BCG, candidiasis	Hepatosplenomegaly, Lymphadenopathy
P311 /M	2		Undefined/CID	18.99	14.92	0.540791	8.19	0.836631	0.642857	CMV pneumonitis	
P72 / M	121		CD40L, Del 347A	31.74	5.35	6.091430	7.31	0.875459	6.920455	Oral candidiasis,	Hepatosplenomegaly
**Auto-inflammatory disorders** (CIAS)											
P234 / M	64		Undefined/periodic fever	24.34	15.07	0.540069	14.28	0.791105	0.683544		
P236 / M	60		Undefined/periodic fever	19.68	14.14	0.546035	9.22	0.813053	0.679012		
P238 / M	86		Undefined/periodic fever	40.81	undetectable	undetectable	undetectable	undetectable	D		
**Phenocopies of PID** (Auto IFN-γ Abs)											
P32 / F	613		Undefined/recurrent NTM	34.38	7.28	1.992047	0.24	12.215801	0.162848	NTM, varicella, Talaromyces (Penicillium) marneffei	Lymphadenopathy
P188 / F	624		Undefined/recurrent NTM	21.91	8.34	1.233055	7.95	0.844993	1.464286	NTM	Lymphadenopathy
P197 / M	675		Undefined/recurrent NTM	20.50	8.9	1.008003	7.39	0.870873	1.157462	NTM	Lymphadenopathy
P362 / M	972		Undefined/recurrent NTM	20.86	12.98	0.561537	7.62	0.859018	0.651163	NTM	
**Congenital defects of phagocyte number, function or both**											
P54 / M	66		STAT1, Thr385Met	23.45	Undetectable	undetectable	undetectable	Undetectable	D	BCG, candidiasis	Hepatosplenomegaly, Lymphadenopathy,

												Hypothyroidism
P22 / M	31		Gp91, Del 1693G	22.25	9.96	0.761076		8.29	0.833536	0.915663	BCG, candidiasis	Splenomegaly
P389 / M	4		Gp91, n. Del846, 847 TG	22.26	11.83	0.595743		6.72	0.918422	0.652174	BCG, aspergillosis	Lung granuloma
P404 / M	3		Gp91, Del exon 1, 2	21.82	17.62	0.534604		9.33	0.811392	0.654321	BCG	Lung granuloma
P437 / M	5		Gp91, n.676C>T, Arg226Stop	19.97	16.85	0.535399		10.41	0.800413	0.668902	BCG, aspergillosis	
P450 / M	14		Gp91, n.1028C>A, p.Thr343Lys	20.13	14.67	0.542175		9.62	0.807814	0.666667	BCG, aspergillosis	Lymphadenopathy
P185 / M	94		Gp91, n.1249G>T, Gly412Val	23.12	11.59	0.607013		9.51	0.808933	0.753086		
P226 / M	3		Gp91, n.497C>T, Del exon 5	22.18	15.78	0.537507		8.74	0.821985	0.658537		
P194 / M	38		Gp91, 1601 Ins G, Fs, stop540	27.32	14.29	0.544795		9.33	0.811392	0.666667	BCG	
P227 / M	39		Undefined /decreased ROS	32.84	9.09	0.949449		1.65	5.089927	0.186640	BCG, aspergillosis	
P363 / M	102		Gp47, del 75,76GT	21.1	15.95	0.537060		10.14	0.802385	0.669329	Aspergillosis	
P326 / M	84		Gp47, del 75,76GT	19.70	16.76	0.535521		13.44	0.791640	0.683544	Aspergillosis	
P98 / M	25		Undefined/neutropenia	22.11	9.59	0.827614		7.51	0.864967	0.965116		
P425 / F	212		Undefined/neutropenia	20.89	11.63	0.605002		16.52	0.790570	0.772152		
P66 / M	14		Undefined/neutropenia	21.30	undetectable	undetectable		15.87	0.790652	zero		
P61 / F	61		Undefined/neutropenia	29.31	13.67	0.550871		7.41	0.870317	0.632184		
P63 / M	122		Undefined/neutropenia	20.13	undetectable	undetectable		15.65	0.790689	zero		
P306 / M	12		Undefined/neutropenia	19.72	14.64	0.542357		9.69	0.806762	0.666667		
P132 / M	87		IFNGR, 818Del 4 nu.	21.94	10	0.754852		7.26	0.878458	0.852273	BCG	Lymphadenopathy
**Combined immunodeficiencies with associated or syndromic features**												
P225 / M	158		WAS, Int(+5)G>A, skip exon 8	19.70	14.42	0.543820		7.20	0.882197	0.613636		Severe atopic dermatitis, lymphoma
P192 / M	5		WAS, Arg13Stop	22.79	13.93	0.548002		9.02	0.816418	0.670732		Atopic dermatitis,
P416 / M	3		WAS, Del 243-250 nu	20.96	18.34	0.534162		10.26	0.801430	0.666511		Atopic dermatitis, ITP
P436 / M	140		WAS, 1023 Ins C	20.11	16.73	0.535564		10.78	0.798102	0.671047		Atopic dermatitis

P154 / M	9		WAS, Arg41Stop	31.89	undetectable	undetectable		7.85	0.848911	zero		Atopic dermatitis
P247 / M	106		WAS, 1023 Ins C	22.30	9.8	0.787770		16.10	0.790618	0.996400		ITP
P247U / M	264		WAS, 1023 Ins C	19.53	16.04	0.536843		8.12	0.838928	0.642857		Atopic dermatitis,
P162 / M	157		SATA3, Gln469Arg	28.10	12.65	0.568748		9.86	0.804946	0.706567		Cutaneous granuloma
P274 / M	82		Undefined / HIES	22.51	17.38	0.534808		8.76	0.82155	0.646341		
P315 / M	134		STAT3, IVS10(-2)A>G, skip exon 11	19.12	14.97	0.540542		9.31	0.811832	0.666667		IBD-like
P178 / M	120		ATM, Arg805Stop, Lys486Fs	23.13	11.12	0.635331		9.04	0.816060	0.780488		CNS lymphoma
P208 / F	266		Undefined / HIES	22.79	14.52	0.543127		10.40	0.800413	0.678559		Atopic dermatitis
**Defects in innate immunity (EAD-ID)**												
P244 / F	40		NEMO exon 4-10 del	21.61	15.88	0.537238		8.99	0.816964	0.658537		Herpes-like dermatitis
P245/ F	1		NEMO exon 4-10 del	23.86	15.97	0.537010		9.24	0.812742	0.666667		Herpes-like dermatitis
P369/ F	2		NEMO exon 4-10 del	21.36	8.28	1.262763		14.22	0.791133	1.594937		Herpes-like dermatitis
P278 / F	78		Undefined/IP	20.63	11.74	0.599751		6.34	0.956993	0.626704		Herpes-like dermatitis
P385 / M	16		Undefined/IP	23.71	15.26	0.539256		8.34	0.832068	0.650602		Herpes-like dermatitis
P332 / F	6		Undefined/IP	20.51	18.17	0.534247		10.37	0.800622	0.667290		Herpes-like dermatitis
P451 / F	1		Undefined/IP	21.95	13.15	0.558418		5.79	1.034294	0.543689		Herpes-like dermatitis
P26 / M	108		Undefined/alopecia	21.60	13.56	0.552248		11.19	0.796202	0.693603		Alopecia
P379 / M	109		Undefined/CMC	31.30	undetectable	undetectable		8.54	0.826677	zero	CMC	
**Diseases of immune dysregulation**												
P296 / M	12		XIAP, Int 5 (+1) G>A	19.25	11.51	0.611205		10.71	0.798482	0.765459	Candidiasis	HLH-like, IBD
P405 / M	2		FOXP3, Met370Leu	19.27	12.9	0.563136		5.86	1.022744	0.550613	Candidiasis	IBD
P182 / M	217		Undefined/HLH	27.01	13.59	0.560208		8.51	0.827439	0.674699		HLH
P214 / F	144		Undefined/HLH	21.10	9.31	0.890617		6.33	0.958151	0.927083		HLH

**Complement deficiencies**												
P351 / F	24		C3, Int 11 (+1) G>T	26.61	13.25	0.556748		9.21	0.813211	0.691358		
**Other unclassified PIDs phenotype**												
P459 / F	22		Undefined/Recurrent infections, lymphoma	20.75	14.01	0.547219		6.19	0.975243	0.561224		Lymphoma
P96 / M	188		Undefined/refractory TB	16.36	9.52	0.842238		9.26	0.812434	1.037037	TB	
P168 / M	2		Undefined/leukocytosis	26.16	12.83	0.564611		9.17	0.813851	0.691358		
P90 / F	28		Undefined/recurrent cellulitis	21.35	12.03	0.587683		6.86	0.906585	0.648352		
P286 / M	269		Undefined/alopecia, albinism and severe atopic dermatitis	23.19	15.95	0.537060		11.52	0.795021	0.675529		Alopecia, albinism, severe atopic dermatitis
P256 / M	34		Undefined/recurrent pneumonia	23.50	14.10	0.546388		7.25	0.879070	0.621552		
P267 / M	8		Undefined/recurrent pneumonia	21.01	16.94	0.535282		7.70	0.855318	0.627907		
P317 / F	504		Undefined/recurrent NTM	19.59	15.36	0.538869		8.85	0.819668	0.658537	NTM	
P249 / M	264		Undefined/recurrent NTM	19.44	13.41	0.554305		9.11	0.815016	0.670732	NTM	
P318 / M	77		Undefined/refractory NTM	22.03	13.96	0.547704		7.31	0.875459	0.625619	NTM	
P322 / M	80		Undefined/recurrent NTM	18.36	15.47	0.538473		9.27	0.812282	0.666667	NTM	

Gray ground meant below 5% of normal range; Pink ground meant over 95% of normal range.

Abbreviations: CVID, common variable immunodeficiency; HIGM, hyper IgM syndrome; THI, transient hypogammaglobulinemia of infancy; NTM, non-tuberculosis mycobacterial infections; HLH, hemophagocytic lympho-histiocytosis; HIGE, hyper IgE recurrent infection syndromes; IP, incontinentia pigmenti; IBD, inflammatory bowel disease; ITP, idiopathic thrombocytopenia purpura.

In the “Predicted Th17/Treg ratio” column, “zero” meant undetectable RT-PCR FOXP3 and “D” meant undetectable RT-PCR both FOXP3 and RORγt.

Supplemental Table 4. The p value in statistical t test and Mann-Whitney comparison of the predicted Treg percentages in each PIDs category*

Category<br>Percentage	Number	**Predominantly B**	**Combined T and B**	**Autoinflammation**	**Phenocopies**	**Phagocyte**	**Syndromic**	**Innate**	**Immne dys**	**Unclassified**
**Healthy controls**	29	**0.0031**	**0.0030**	**0.0294**	**0.0127**	0.5828	**0.0155**	0.0583	0.2950	**0.0356**
1.139 ± 0.08409		**<0.0001**	**<0.0001**	**0.0055**	**0.2357**	**<0.0001**	**<0.0001**	**0.0020**	0.3076	**0.0006**
**Predominantly B**	19		0.1144	0.0912	**0.0236**	0.4021	0.8099	0.5177	0.3773	0.4758
0.8046 ± 0.04744			**0.0237**	0.0621	**0.0387**	0.4556	0.6265	0.9680	0.3107	0.5469
**Combined T and B**	9			0.7139	0.1149	0.2933	0.0830	0.0907	0.1749	0.0586
0.6308 ± 0.1196				1.0000	**0.0196**	0.0851	**0.0429**	**0.0044**	**0.0336**	**0.0088**
**Autoinflammation**	3				0.3907	0.4432	**0.0317**	0.0564	0.1752	**0.0288**
0.5347 ± 0.2674					0.0571	0.2712	**0.1297**	0.1000	0.1143	**0.0430**
**Phenocopies**	4					**0.0488**	0.0787	0.1387	0.3629	0.0973
3.698 ± 2.839						**0.0470**	**0.0181**	0.0755	0.6857	0.2672
**Phagocyte**	19						0.5314	0.6567	0.8429	0.6195
1.005 ± 0.2312							0.8711	0.4606	0.2087	0.0707
**Syndromic**	12							0.2203	**0.0200**	0.0861
0.8193 ± 0.0075								0.6441	0.2030	0.6481
**Innate**	9								0.3837	0.9733
0.8520 ± 0.02826									0.4140	0.6485
**Immne dys**	4									0.2359
0.9017 ± 0.05323										0.4727
**Unclassified**	11									
0.8509 ± 0.01643										

* The value expressed by mean ± standard error of mean (SEM). Undetectable *FOXP3* production by RT-PCR was designed as “0”. The “complement” category was omitted because of only one case was enrolled in this analysis. PIDs: primary immunodeficiency diseases. Bold texts indicated significance ($p<0.05$) by t-test (in the upper lane) or Mann-Whitney test (in the lower lane)

Supplemental Table 5. The p value in statistical t test and Mann-Whitney comparison of the predicted Treg percentages in each PIDs category*

Category<br>Percentage	Number	**Predominantly B**	**Combined T and B**	**Autoinflammation**	**Phenocopies**	**Phagocyte**	**Syndromic**	**Innate**	**Immne dys**	**Unclassified**
**Healthy controls**	29	**<0.0001**	0.6253	**0.0095**	0.8579	**<0.0001**	**<0.0001**	**0.0016**	0.0513	**0.0040**
1.1531 ± 0.8893		**<0.001**	**0.0005**	**0.0055**	0.8469	**<0.0001**	**<0.0010**	**0.0002**	**0.0088**	**<0.0001**
**Predominantly B**	19		0.3835	0.1129	**0.0011**	0.4156	0.4042	0.8575	0.5337	0.8719
0.5870 ± 0.04705			**0.0365**	0.2317	**0.0168**	0.6935	0.3510	0.4458	0.1558	0.9143
**Combined T and B**	9			0.6070	0.8302	0.3165	0.4280	0.5414	0.7528	0.4991
0.9770 ± 0.6452				0.8636	**0.0336**	0.1096	0.1551	0.2581	0.0336	**0.0483**
**Autoinflammation**	3				0.0817	0.3395	0.2424	**0.0279**	0.1591	0.0511
0.3620 ± 0.1810					0.0571	0.4728	0.5636	0.6000	0.0571	0.2130
**Phenocopies**	4					**0.0013**	**0.0027**	**0.0279**	0.1300	**0.0034**
1.199 ± 0.2990						**0.0134**	**0.0130**	**0.0336**	0.2000	**0.0158**
**Phagocyte**	19						0.9866	0.6962	0.3462	0.5299
0.5243 ± 0.05991							0.6850	0.8633	0.1335	0.7145
**Syndromic**	12							0.6971	0.2206	0.4120
0.5257 ± 0.05227								0.8312	0.0602	0.3402
**Innate**	9								0.6187	0.9422
0.5690 ± 0.1061									0.0755	0.6485
**Immne dys**	4									0.2319
0.6563 ± 0.07898										0.0584
**Unclassified**	11									
0.5763 ± 0.02698										

* The value expressed by mean ± standard error of mean (SEM). Undetectable *RORγt* production by RT-PCR was designed as “0”. The “complement” category was omitted because of only one case was enrolled in this analysis. PIDs: primary immunodeficiency diseases. Bold texts indicated significance ($p<0.05$) by t-test (in the upper lane) or Mann-Whitney test (in the lower lane).

Supplemental Table 6. The p value in statistical comparison of the predicted ratio of Th17/Treg in each PIDs category*

Category<br>Ratio	Number	**Predominantly B**	**Combined T and B**	**Autoinflammation**	**Phenocopies**	**Phagocyte**	**Syndromic**	**Innate**	**Immne dys**	**Unclassified**
**Healthy controls**	29	**0.0012**	0.1641	0.1712	0.3747	**0.0006**	**0.0040**	0.0556	0.0978	**0.0024**
1.044 ± 0.06688		**<0.0001**	**0.0192**		0.6003	**<0.0001**	**<0.0001**	**0.0016**	**0.0221**	**<0.0001**
**Predominantly B**	17		0.1124	0.6540	0.3801	0.5032	0.6277	0.7184	0.9868	0.3691
0.7281 ± 0.03433			0.8610		0.8228	0.8288	0.7067	0.3512	0.6869	0.1513
**Combined T and B**	6			0.6071	0.5364	0.1123	0.1979	0.3122	0.4723	0.1886
1.713 ± 1.042					0.9143	0.4389	0.6152	0.1812	0.7619	0.1193
**Autoinflammation**	2				0.7003	0.9345	0.7789	0.7537	0.7059	0.9925
0.6813 ± 0.002266										
**Phenocopies**	4					0.2986	0.3762	0.7181	0.6781	0.3198
0.8589 ± 0.2863						0.8873	0.7441	0.9333	0.8857	0.5569
**Phagocyte**	16						0.8347	0.4941	0.6890	0.8540
0.6915 ± 0.04216							0.9606	0.4082	0.6033	0.2461
**Syndromic**	11							0.5910	0.7184	0.6413
0.7036 ± 0.03194								0.5088	0.6447	0.3245
**Innate**	8								0.8583	0.4656
0.7628 ± 0.1199									0.4606	0.7725
**Immne dys**	4									0.5367
0.7295 ± 0.07923										0.4727
**Unclassified**	11									
0.6804 ± 0.03724										

* The value expressed by mean ± standard error of mean (SEM). Undetectable *FOXP3* or *RORγt* production by RT-PCR was omitted because of logically mathematic consideration. The "complement" category was omitted because of only one case was enrolled in this analysis. PIDs: primary immunodeficiency diseases.

Bold texts indicated significance ($p<0.05$) by t-test (in the upper lane) or Mann-Whitney test (in the lower lane).

Supplemental Table 7. The p value in statistical comparison of the predicted ratio of Th17/Treg in each PIDs category*

Disease<br>Percentage	Number	**BTK**	**CVID**	**SCID & CID**	**CGD**	**WAS**
**Healthy controls**<br>1.044 ± 0.06688	29	**0.0009**<br>**0.0002**	**0.0057**<br>**<0.0001**	**<0.0001**<br>**<0.0001**	**0.0006**<br>**0.0001**	**0.0014**<br>**0.0004**
**BTK**<br>0.6660 ± 0.06139	6		0.6331<br>0.1079	0.0856<br>0.0593	0.5661<br>0.9578	0.5762<br>0.5338
**CVID**<br>0.8553 ± 0.7256	6			0.0812<br>**0.0104**	0.6559<br>0.1728	0.4231<br>0.1893
**SCID & CID**<br>0.6710 ± 0.008971	8				**0.0203**<br>**0.0117**	0.2573<br>0.1893
**CGD**<br>0.6989 ± 0.02578	10					0.3576<br>0.6009
**WAS**<br>0.7102 ± 0.05796	7					

* The value expressed by mean ± standard error of mean (SEM). Undetectable *FOXP3* or *RORγt* production by RT-PCR was omitted because of logically mathematic consideration.
PIDs: primary immunodeficiency diseases.
Bold texts indicated significance ($p<0.05$) by t-test (in the upper lane) or Mann-Whitney test (in the lower lane).

Supplemental Table 8. The correlation of the phenotypes of opportunistic infections, autoimmune disorders to the predicted Th17 cells, Treg cells and the Th17/Treg ratio

	Patient number				
Subgroup	**Opportunistic infection**			**Autoimmune disorders**	
	33	58		42	49
	With	Without		With	Without
**Low Th17**	26	52	**Low Treg**	30	38
**Non-low Th17**	7	6	**Non-low Treg**	12	11
Chi-squire p value		0.1544			0.5029
**Low Th17/Treg**	26	45		31	40
**Non-low Th17/Treg**	7	13		11	9
Chi-squire p value		0.8941			0.8072
**Low Th17 & low Treg**	20	42		25	27
**Other**	13	16		17	22
Chi-squire p value		0.2452			0.3690
**Low Th17 & low Th17/Treg**	24	45		30	39
**Other**	9	13		12	10
Chi-squire p value		0.6027			0.3646
**Low Treg & low Th17/Treg**	18	36		21	33
**Other**	17	20		21	16
Chi-squire p value		0.2245			0.0931

Supplemental Table 9. The correlation of the phenotypes of opportunistic infections, autoimmune disorders to the predicted Th17 cells, Treg cells and the Th17/Treg ratio by binary logistic regression

	**Opportunistic infections**		**Autoimmune disorders**	
	**95% of C.I.**	***p* value**	**95% of C.I.**	***p* value**
**Predicted Th17 percentage**	0.647-82.257	0.108	0.727-1.708	0.494
**Predicted Treg percentage**	0.160-52.641	0.471	0.772-1.708	0.494
**Predicted T17/Treg ratio**	0.586-4.686	0.341	0.390-32.990	0.330

Supplemental Figure 1. Real-time PCR was amplified for FOXP3 (Hs01085834_m1) and RORγt (Hs01076112_m1) with GAPDH (Hs99999905_m1) as the internal control. Representative demonstrations in patients were P4, P90, P185, P244 and P278 (RFU intensity of CT =249.82).

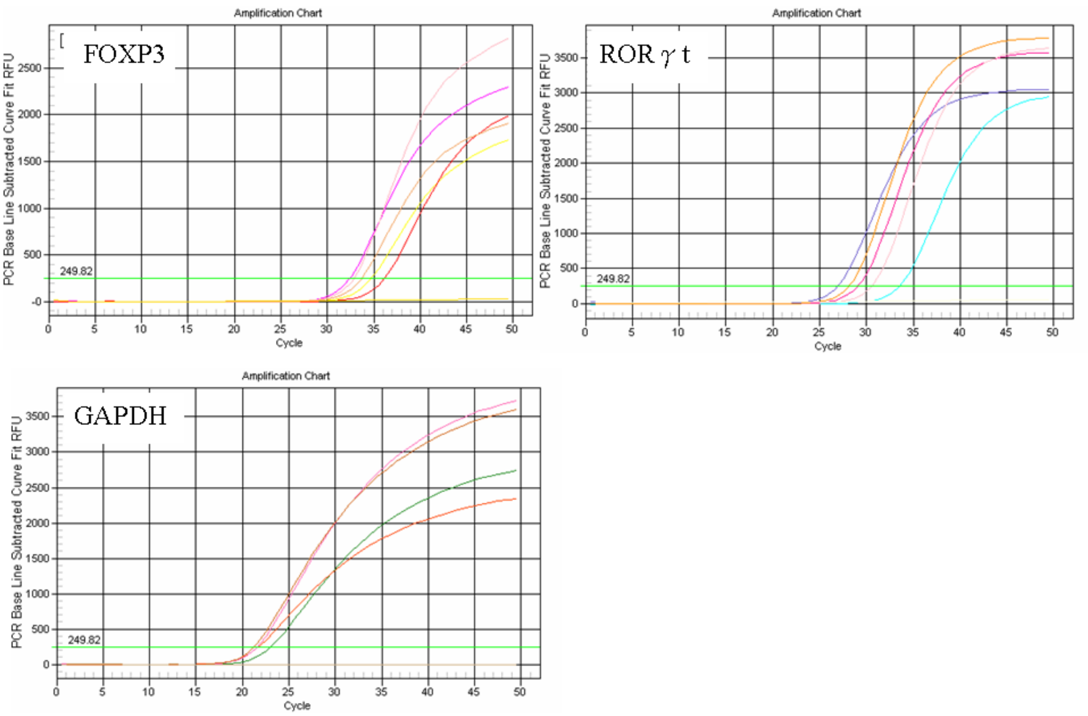

Supplemental Figure 2. The predicted Treg percentages in BTK, CVID, CID, CGD and WAS patients and healthy controls were calculated and analysis by t test (in the upper lane) and Mann-Whitney test (in the lower lane) meaning significance if $p < 0.05$. The values were expressed by mean ± standard error of mean (SEM). Undetectable *FOXP3* production by RT-PCR was designed as "0". Compared to the controls, these patients had significantly lower Treg cell percentages. Thick numbers indicated significance ($p<0.05$).

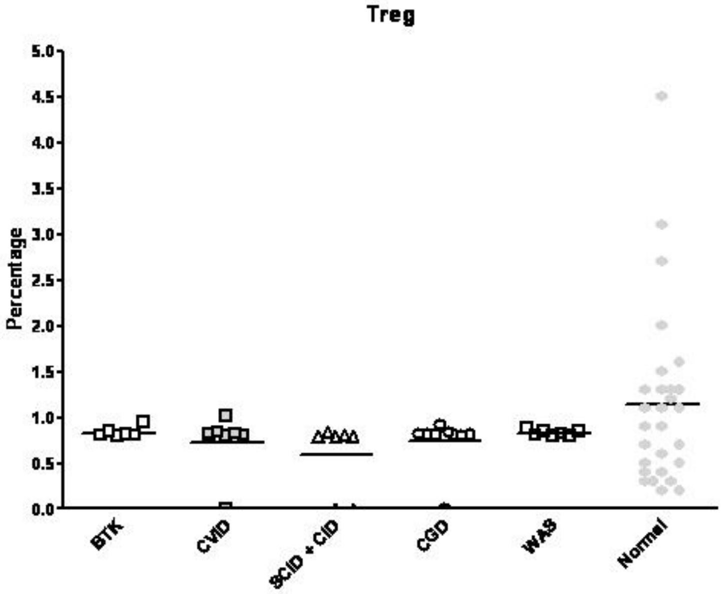

Disease<br>Percentage	Number	BTK	CVID	SCID & CID	CGD	WAS
**Healthy controls**	29	0.0979	**0.0187**	**0.0029**	**0.0274**	0.0671
1.139 ± 0.08409		**0.0020**	**0.0006**	**<0.0001**	**<0.0001**	**0.0002**
**BTK**	6		0.4518	0.1568	0.4199	0.7641
0.8332 ± 0.02416			0.7546	**0.0200**	0.8016	1.0000
**CVID**	6			0.4427	0.9261	0.4477
0.7342 ± 0.1077				0.0830	1.0000	0.7789
**SCID & CID**	8				0.3181	0.1352
0.6002 ± 0.1311					**0.0352**	**0.0289**
**CGD**	10					0.4223
0.8208 ± 0.01144						0.6507
**WAS**	7					
0.8252 ± 0.01251						

Supplemental Figure 3. The predicted Th17 percentages in BTK, CVID, CID, CGD and WAS patients and healthy controls were calculated and analysis by t test (in the upper lane) and Mann-Whitney test (in the lower lane) meaning significance if $p < 0.05$. The values were expressed by mean ± standard error of mean (SEM). Undetectable *RORγt* production by RT-PCR was designed as “0”. Compared to the controls, these patients had significantly lower Th17 cell percentages. Thick numbers indicated significance $p < 0.05$.

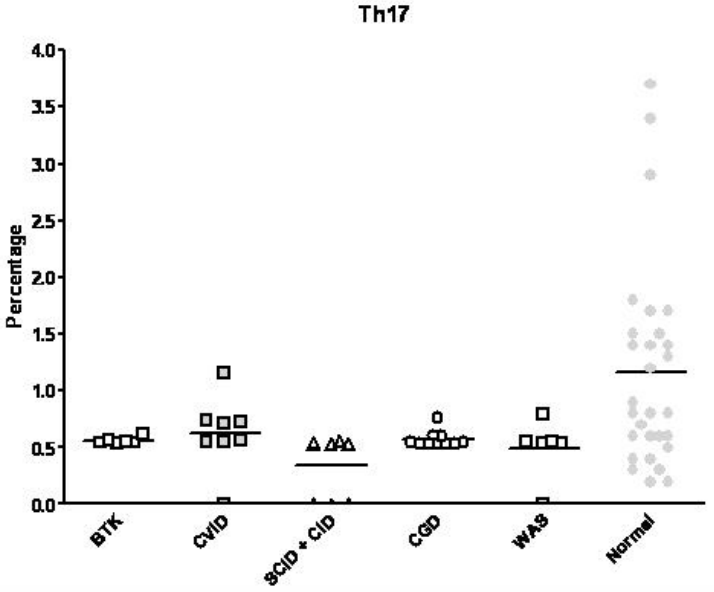

Disease<br>Percentage	Number	BTK	CVID	SCID & CID	CGD	WAS
**Healthy controls**	29	**0.0049**	**0.0057**	**<0.0001**	**0.0006**	**0.0014**
1.151 ± 0.08409		**0.0002**	**<0.0001**	**<0.0001**	**0.0001**	**0.0004**
**BTK**	6		0.6331	0.0856	0.5661	0.5762
0.8046 ± 0.04744			0.1079	0.0593	0.9578	0.5338
**CVID**	6			0.0812	0.6559	0.4231
0.6308 ± 0.1196				**0.0104**	0.1728	0.1893
**SCID & CID**	8				**0.0203**	0.2573
0.5347 ± 0.2674					**0.0117**	0.1893
**CGD**	10					0.3576
0.5731 ± 0.2839						0.6009
**WAS**	7					
1.0050 ± 0.2312						
